# Neuropsychological rehabilitation in executive deficits resulting from alcohol use disorder: systematic review of literature

**DOI:** 10.3389/fpsyg.2026.1805577

**Published:** 2026-04-29

**Authors:** Sónia Ferreira, Ana Virgolino, Samuel Pombo, Leonor Bacelar-Nicolau, Enrique Vásquez-Justo, Cristina Ribeiro

**Affiliations:** 1 Unidade de Tratamento e Reabilitação de Alcoólicos (UTRA), na Unidade de Local de São José, Lisbon, Portugal; 2 Faculdade de Medicina, Universidade de Lisboa, Lisbon, Portugal; 3 Instituto de Saúde Ambiental, Faculdade de Medicina, Universidade de Lisboa, Lisbon, Portugal; 4 Laboratório Associado TERRA, Faculdade de Medicina, Universidade de Lisboa, Lisbon, Portugal; 5 Serviço de Psiquiatria e Saúde Mental do Hospital de Santa Maria/Clinica Universitária de Psiquiatria e Psicologia Médica da Faculdade de Medicina, Universidade de Lisboa, Lisbon, Portugal; 6 Instituto de Medicina Preventiva e Saúde Pública & Área Disciplinar Autónoma de Bioestatística (Laboratório de Biomatemática), Lisbon, Portugal; 7 Escola Superior de Educação de Fafe, Braga, Portugal; 8 Universidad Camilo José Cela: Villafranca del Castillo, Madrid, Spain; 9 Instituto de Medicina Preventiva e Saúde Pública, Clínica Universitária de Medicina Geral e Familiar, Faculdade de Medicina, Universidade de Lisboa, Lisbon, Portugal

**Keywords:** alcoholism, executive functions, flexibility, inhibition, neuropsychological rehabilitation, planning, working memory

## Abstract

**Objectives:**

In recent years, there has been a growing interest in neuropsychological rehabilitation for alcohol use disorders. However, doubts persist regarding its impact on the recovery of executive functions. This systematic literature review summarizes the existing empirical evidence on the association between neuropsychological rehabilitation and the recovery of executive functions, including working memory, inhibition, flexibility, and planning, in individuals with alcohol use disorders.

**Methods:**

This review (registration number, Prospero CRD42023393730) was conducted in accordance with the PRISMA guidelines. The PubMed, Web of Science, Scopus, and Cochrane databases were searched. Only randomized clinical trials were included. A narrative analysis of the results was undertaken. The Cochrane Collaboration tool ROB-2 was used to assess risk of bias.

**Results:**

Eight randomized controlled trials with 356 participants were considered eligible. The results indicated that neuropsychological rehabilitation can positively impact the recovery of executive functions in individuals with alcohol use disorders. Some studies also found improvements in non-cognitive domains with this type of intervention, such as the compulsion to consume alcohol, psychological wellbeing, and relapse prevention. Most studies assessed efficacy after 1 month.

**Conclusion:**

Neuropsychological rehabilitation promotes greater efficacy in recovering executive functions in alcohol use disorder treatment.

**Systematic review registration:**

https://www.crd.york.ac.uk/PROSPERO/view/CRD42023393730.

## 1 Introduction

Between 50 and 80 percent of individuals with alcohol use disorder (AUD) exhibit cognitive impairments related to alcohol consumption (Caneva et al., 2020), particularly in executive functions (EFs) (Crespi et al., 2019). EFs are higher-order cognitive skills that facilitate control over thoughts, behaviors, and emotions; adaptation to new situations; and the planning and organization of actions to achieve specific objectives, among other things (Verdejo-Garcia, 2017; Bechara, 2005). EFs are a multidimensional construct, consisting of different components. According to some theoretical models, such as that of Miyake et al. (2000) and Friedman and Miyake (2017), there are three central components: working memory, cognitive flexibility, and inhibition. Despite their specificities, these components are interdependent. Other, more comprehensive models suggest that EFs are not only cognitive processes but are also strictly associated with emotional regulation, planning, and decision-making (Bechara, 2005). These components are considered superior, yet strictly dependent on the former. Damage to these functions occurs at the level of basic components, with greater vulnerability in working memory (WM) and inhibition, as well as at the level of more complex EFs (Loeber et al., 2010; Brooks et al., 2020). This damage is associated with global cerebral atrophy, particularly in the frontal lobes (Oscar-Berman and Marinković, 2007; Sullivan and Pfefferbaum, 2019), the limbic system, and the cerebellum, as well as in connections between these structures (Friedman and Miyake, 2017). It is also related to consumption patterns and intra-individual variables. Literature often analyzes EFs as a unified whole, which hinders understanding of the specific deficits associated with AUD (Friedman and Miyake, 2017). This review seeks to overcome this limitation by clearly defining the components to be analyzed (basic: WM, inhibition, and flexibility) and complex (planning). WM refers to the ability to temporarily retain and manipulate information to perform cognitive tasks (Baddeley and Hitch, 1974). Cognitive flexibility is the ability to change strategies, adapt to new rules, or switch between different tasks or ideas (Dajani and Uddin, 2015). Inhibition refers to the ability to control impulses, resist distracting stimuli, and avoid inappropriate automatic responses. Planning, in turn, refers to the ability to anticipate steps, organize actions, and define strategies to achieve a goal (Malloy-Diniz et al., 2014).

Impairment of these components’ functioning can compromise adherence to treatment (Oscar-Berman and Marinković, 2007; Kopera et al., 2012; Le Berre et al., 2017). Although abstinence leads to improvements in these functions, some deficits may persist (Bates et al., 2023; Romero-Martínez et al., 2020). Therefore, it is necessary to foster this improvement to facilitate the alcohol rehabilitation process. This can lead to difficulties resisting cravings, engaging in risky consumption, regulating emotions (Bates et al., 2023; Romero-Martínez et al., 2020; Pitel et al., 2009; Petit et al., 2017), and using maladaptive coping strategies (Andó et al., 2012), all of which affect the rehabilitation of these individuals. Recovery of these functions is essential (Verdejo-Garcia et al., 2019) so that traditional treatments for alcohol abuse can successfully promote alcohol abstinence and facilitate social and personal reintegration.

Neuropsychological rehabilitation (NR) is increasingly recognized as an effective intervention for addressing this problem (Verdejo-Garcia, 2017; Friedman and Miyake, 2017; Verdejo-Garcia et al., 2019; Diamond, 2013). NR consists of neuropsychological interventions that aim to improve affected skills and develop compensatory and adaptive strategies. These interventions improve cognitive, emotional, and social impairments (Frías-Torres et al., 2018) and enhance maintained skills, thereby improving functionality and quality of life (Diamond, 2013; Bates et al., 2023; Duka and Stephens, 2014; Frías-Torres et al., 2018; Hayes et al., 2016). NR uses different models, some of which focus on training specific functions. These models aim to improve, reinforce or repair skills through repetitive exercises with increasing degrees of complexity using software and/or pencil-and-paper exercises (Wilson, 1989; Gamito et al., 2014a; Fals-Stewart and Lam, 2010). It is essential that the skills developed through NR be applicable to other situations that require the same or different skills (transfer effect) and that they improve functionality, making individuals more capable in their daily routines (Fals-Stewart and Lam, 2010; Roehrich and Goldman, 1993). In this sense, integrative models that consider cognitive, social, and affective aspects are recognized as facilitating comprehensive rehabilitation (Verdejo-Garcia et al., 2023). However, the variables that characterize these programs and how they promote or hinder the effectiveness must be explored more effectively, considering factors that may affect the results, such as individual and consumption variables.

Regarding the effectiveness of these interventions, although there is evidence on the effects of NR on AUD (Snider et al., 2018; Snider et al., 2018; Kumari and Kumar, 2020), the studies do not agree on their results (Nixon and Lewis, 2019; Caetano et al., 2021; Jones et al., 2018), particularly regarding the impact on consumption (Rupp et al., 2012; Jones et al., 2018; Houben et al., 2011), or on other areas of patients’ lives. This underscores the need for a better understanding of this topic, the focus of this review.

Previous systematic reviews in this area did not focus exclusively on AUD or EFs (Caetano et al., 2021) and included other interventions, such as medication or non-invasive brain stimulation (Spagnolo et al., 2020). This limits the assessment of the role of NR. Others failed to control for confounding variables, such as brain injuries or psychiatric conditions (Nixon and Lewis, 2019). This review’s innovation lies in its focus on EFs and restriction of the analysis to AUD only. Given its specificities, such as the neurophysiological effects of alcohol and its impact on cognition, an analysis of AUD is essential. It is also necessary to adapt treatment to the needs resulting from alcohol consumption and the damage it causes (Levine and Pivneva, 2010). Focusing on EFs is important because deficits in these areas are highly prevalent in this population and are linked to other affected skills (Oscar-Berman and Marinković, 2007). This review aims to provide a more comprehensive overview of the various structural elements of NR, including the use of different rehabilitation strategies and tasks, program duration, and the frequency and number of training sessions. It also describes when and how evaluations and progress monitoring are conducted. The review summarizes the evidence for the efficacy of NR in studied components such as inhibition, WM, flexibility, and planning, in AUD, as well as its transfer effect to non-cognitive domains, such as alcohol consumption. The goal is to contribute to the standardization of interventions in this area. The research question is as follows: How effective is NR in recovering executive deficits (e.g., inhibition, WM, flexibility, and planning) in AUD?

## 2 Methods

This review was conducted in accordance with the Cochrane (Higgins et al., 2024) and Preferred Reporting Items for Systematic Reviews and Meta-Analyses (PRISMA) guidelines for systematic reviews (Moher et al., 2015). This review is registered with the International Prospective Register of Systematic Reviews (registration number CRD42023393730). Although the protocol was published, some inclusion criteria were reformulated to achieve greater rigor in data analysis (Ferreira et al., 2024).

### 2.1 Search strategy and selection criteria

The PubMed, Web of Science, Scopus, and Cochrane electronic databases were consulted. The search was conducted on May 5th, 2023 and adjusted in April 2025. No time limitations were applied, and free text keywords and Medical Subject Headings (MeSH) terms were used wherever possible. The keywords were defined based on a prior analysis of relevant research on AUD, executive functions, and neuropsychological rehabilitation (central concepts in the research question). This analysis included a review of reference articles in the field, systematic literature reviews, and clinical trials to identify the terms most frequently used in research to characterize this population, executive functions, and this type of intervention. Based on this preliminary review, the keywords were grouped into three categories: one relating to alcoholism, one relating to cognitive domains, and one relating to neurocognitive training. The research team, composed of professionals with clinical and scientific experience in the field, made the final selection by consensus, ensuring that the research strategy rigorously and comprehensively reflected the conceptual framework of the review.

Only studies published in English, Spanish, French, or Portuguese were included in the search (see Table 1 for the search strategy).

**Table 1:** Search strategy.

Electronic Databases	Search Strategy
Web of science	AB = (“alcohol” OR “alcohol use disorder” OR “alcoholism” OR “alcohol dependence syndrome” OR “alcoholic dependence” AND “neuropsychological rehabilitation program” OR “neuropsychological rehabilitation” OR cognitive train” OR “cognitive remediation” OR “cognitive remediation therapy” OR “cognitive rehabilitation” OR “cognitive rehabilitation program” OR “cognitive stimulation” OR “executive train” AND “executive function” OR “cognitive function” OR “executive deficit” OR “Inhibition” OR “working memory” OR “Plan” OR “cognitive flexibility”)
Scopus	ALL (“alcohol” OR “álcool” OR “alcool” OR “alcohol use disorder” OR “perturbação por uso de álcool” OR “trastorno por consumo de alcohol” OR “trouble lié à la consommation d’alcool” OR “trouble d’abus de consommation d’alcool” OR “problème d’abus de consommation d’alcool” OR “alcoolismo” OR “alcoholism” OR “alcoolisme” OR “alcohol dependence syndrome” OR “síndrome de dependencia de alcohol” OR “síndrome de dependência alcoólica” OR “sindrome de dependência alcoólica” OR “syndrome de dépendance à l’alcool” OR “alcoholic dependence” OR “dependencia alcohólica” OR “dependência alcoólica” OR “dépendance alcoolique”) AND (“neuropsychological rehabilitation program” OR “programa de reabilitação neuropsicológica” OR “programa de rehabilitación neuropsicológica” OR “programme de réhabilitation neuropsychologique” OR “neuropsychological rehabilitation” OR “reabilitação neuropsicológica” OR “rehabilitación neuropsicológica” OR “réhabilitation neuropsychologique” OR “cognitive train*” OR “trein* cognitivo” OR “entrenamiento cognitive” OR “entraînement cognitive” OR “cognitive remediation” OR “remediação cognitiva” OR “remediación cognitive” OR “remédiation cognitive” OR “cognitive remediation therapy” OR “terapia de remediação cognitiva” OR “terapia de remediación cognitiva” OR “thérapie de remédiation cognitive” OR “cognitive rehabilitation” OR “reabilitação cognitive” OR “rehabilitación cognitive” OR “rehabilitation cognitive” OR “réhabilitation cognitive” OR “cognitive rehabilitation program” OR “programa de reabilitação cognitiva” OR “programa de rehabilitación cognitive” OR “programme de réhabilitation cognitive” OR “cognitive stimulation” OR “estimulação cognitiva” OR “estimulación cognitive” OR “stimulation cognitive” OR “neurocognitive train*” OR “treino neurocognitivo” OR “entrenamiento neurocognitive” OR “entraînement neurocognitive” OR “executive train” OR “treino executivo” OR “entrenamiento ejecutivo” OR “entraînement exécutif”) AND (“executive function” OR “funç* executiva” OR “funcione* ejecutiva” OR “fonction* executive” OR “cognitive* function” OR “funç* cognitiva” OR “funcione* cognitiva” OR “fonction* cognitive” OR “executive deficit” OR “défice* executivo*” OR “déficit* ejecutivo” OR “déficit* exécutif” OR “Inhibition” OR “inibição” OR “inhibición” OR “inhibition” OR “working memory” OR “memória de trabalho” OR “memoria de trabajo” OR “mémoire de travail” OR “Plan*” OR “planeamento” OR “planificación” OR “planification” OR “cognitive flexibility” OR “flexibilidade cognitiva” OR “flexibilidad cognitive” OR “flexibilité cognitive”)
Pubmed	Alcohol[MeSH Terms]) OR (alcohol[Title/Abstract])) OR (álcool[Title/Abstract])) OR (alcool[Title/Abstract])) OR (alcohol use disorder[Title/Abstract])) OR (perturbação por uso de álcool[Title/Abstract])) OR (trastorno por consumo de alcohol[Title/Abstract])) OR (trouble lié à la consommation d’alcool[Title/Abstract])) OR (trouble d’abus de consommation d’alcool[Title/Abstract])) OR (problème d’abus de consommation d’alcool[Title/Abstract])) OR (alcoholism[MeSH Terms])) OR (alcoholism[Title/Abstract])) OR (alcoolismo[Title/Abstract])) OR ((alcoolisme[Title/Abstract])) OR (alcolismo[Title/Abstract])) OR (alcohol dependence syndrome[Title/Abstract])) OR (síndrome de dependência alcoólica[Title/Abstract])) AND (syndrome de dépendance à l’alcool[Title/Abstract])) OR (alcoholic dependence[Title/Abstract])) OR (dependencia alcohólica[Title/Abstract])) OR (dependência alcoólica[Title/Abstract])) OR (dépendance alcoolique[Title/Abstract])) AND ((neuropsychological rehabilitation program[Title/Abstract])) OR (programa de reabilitação neuropsicológica[Title/Abstract])) OR (programa de rehabilitación neuropsicológica[Title/Abstract])) OR ((programme de réhabilitation neuropsychologique[Title/Abstract])) OR (neuropsychological rehabilitation[Title/Abstract])) OR (reabilitação neuropsicológica[Title/Abstract])) OR (rehabilitación neuropsicológica[Title/Abstract])) OR (réhabilitation neuropsychologique[Title/Abstract])) OR (cognitive training[Title/Abstract])) OR cognitive training[MeSH Terms]) OR (formation cognitive[Title/Abstract])) OR (cognitive remediation[MeSH Terms])) OR (cognitive remediation[Title/Abstract])) OR (remediação cognitiva[Title/Abstract])) OR (remediación cognitive[Title/Abstract])) OR (remédiation cognitive[Title/Abstract])) OR (cognitive remediation therapy[Title/Abstract])) OR (terapia de remediação cognitiva[Title/Abstract])) OR (terapia de remediación cognitiva[Title/Abstract])) OR (thérapie de remédiation cognitive[Title/Abstract])) OR (cognitive rehabilitation[Title/Abstract])) OR (reabilitação cognitiva[Title/Abstract])) OR (rehabilitación cognitive[Title/Abstract]))) OR (réhabilitation cognitive[Title/Abstract])) OR (cognitive rehabilitation program[Title/Abstract])) OR (programa de reabilitação cognitiva[Title/Abstract])) OR (programa de rehabilitación cognitive[Title/Abstract])) OR (programme de réhabilitation cognitive[Title/Abstract])) OR (cognitive stimulation[Title/Abstract])) OR (estimulação cognitiva[Title/Abstract])) OR (estimulación cognitive[Title/Abstract])) OR (stimulation cognitive[Title/Abstract])) OR (neurocognitive training[Title/Abstract])) OR (treino neurocognitivo[Title/Abstract])) OR (entraînement neurocognitive[Title/Abstract])) OR (training neurocognitivo[Title/Abstract])) OR (executive training[Title/Abstract])) OR (treino executivo[Title/Abstract])) OR (entrenamiento ejecutivo[Title/Abstract])) OR (entraînement exécutif[Title/Abstract])) AND ((executive function[Title/Abstract])) OR (função executiva[Title/Abstract])) OR (funcione ejecutiva[Title/Abstract])) OR (fonction executive[Title/Abstract])) OR (cognitive function[Title/Abstract])) OR (função cognitiva[Title/Abstract]))) OR (funcione cognitive[Title/Abstract])) OR (fonction cognitive[Title/Abstract])) OR (executive deficit[Title/Abstract])) OR (défice executivo[Title/Abstract])) OR (déficit ejecutivo[Title/Abstract])) OR (déficit exécutif[Title/Abstract])) OR (inhibition[Title/Abstract])) OR (inibição[Title/Abstract])) OR (inhibición[Title/Abstract])) OR (inhibition[Title/Abstract])) OR (working memory[Title/Abstract])) OR (memória de trabalho[Title/Abstract])) OR (memoria de trabajo[Title/Abstract])) OR (mémoire de travail[Title/Abstract])) (planning[Title/Abstract])) OR (planeamento[Title/Abstract])) OR (planificación[Title/Abstract])) OR (planification[Title/Abstract])) OR (cognitive flexibility[Title/Abstract])) OR (flexibilidade cognitiva[Title/Abstract])) OR (flexibilidad cognitive[Title/Abstract])) OR (flexibilité cognitive[Title/Abstract]))
Cochrane	(alcohol OR alcohol use disorder OR alcoholism OR alcohol dependence syndrome OR alcoholic dependence) AND (neuropsychological rehabilitation program OR neuropsychological rehabilitation OR cognitive train OR cognitive remediation OR cognitive remediation therapy OR cognitive rehabilitation OR cognitive rehabilitation program OR cognitive stimulation OR executive train) AND (executive function OR cognitive function OR executive deficit OR Inhibition OR working memory OR Plan OR cognitive flexibility)

To identify additional relevant literature, we consulted the reference lists from the selected articles systematic reviews and meta-analyses, as well as those of other relevant articles.

The titles of the documents found in the three databases were extracted, and duplicates were removed. In cases of retraction or correction, the most recent version of each article was used. Two authors independently evaluated the titles and abstracts to select the articles to be included. In cases of disagreement, a third researcher was consulted to reach a consensus.

The inclusion criteria were defined using the PICO (Population, Intervention, Comparison, and Outcome) framework (Sterne et al., 2019), which is presented in Table 2. due to the broad and multidimensional nature of the concept of executive functions (EFs) is very broad and multidimensional, a clear operational definition was chosen that centered on the core components of EFs (WM, cognitive flexibility, and inhibition) and a higher component: planning. Studies that assessed at least one of these specific components were included, while those assessing cognitive deficits of major neurological origin were excluded, to ensure that alterations in these components are associated with PUA and not with other neurological conditions.

**Table 2:** Inclusion criteria based on the PICO (Population, Intervention, Comparison, and Outcome) framework.

PICO framework	Inclusion criteria	Exclusion criteria
Population	Adults 18 years of age or older; Abstinent; No brain damage, whether caused by alcohol or not.	Studies with participants over 65 years of age only; Participants with brain damage (e.g., head injuries, brain tumors, cerebrovascular accidents, convulsions, loss of consciousness, major neurocognitive disorder, Wernicke-Korsakoff syndrome, or alcoholic dementia).
Intervention	Randomized controlled trials on NR programs integrating the following EFs: inhibition, WM, cognitive flexibility, and planning; Programs that use resources such as pencils, paper, or technology (e.g., computerized training or mobile devices); Clinical context—hospitalization or therapeutic community.	Studies with transcranial magnetic stimulation (TMS); Virtual reality (VR).
Comparison	Usual treatment for AUD; Some type of NR with characteristics (techniques and frequency) that differ from the experimental group; Without any type of intervention.	Outpatient; Non-clinical context.
Outcome	Main outcome: changes in the components of the executive functions studied, as measured by cognitive tests. This considers data from the baseline and the last available follow-up. Other outcomes: changes in other untrained cognitive domains (transfer effect), as measured by cognitive tests.	

It is important to explain why these exclusion criteria were chosen. Studies that only included participants over 65 years of age were excluded due to the increased risk of cognitive deterioration associated with aging. Studies involving transcranial magnetic stimulation (TMS) and virtual reality (VR) were also excluded because these approaches differ in their mechanisms of action, therapeutic objectives, and confounding factors compared to traditional RN programs. TMS uses magnetic fields that directly modulate cortical excitability and produce immediate neurophysiological effects independent of cognitive training. In contrast, NR is based on the concept of learning through repetition, where neuroplasticity is associated with practice. On the other hand, virtual reality VR uses interactive, immersive, and multisensory virtual environments that promote emotional and motivational engagement, contributing to improved cognitive performance without specific training. This can generate confounding factors when compared to traditional NR and compromise the interpretation of its effectiveness (Lefaucheur et al., 2020; Cicerone et al., 2000). VR was eliminated since traditional NRs differ from it, even those that use technology. Traditional software provides tasks in simple, accessible virtual environments with a lower degree of immersion and focuses on practicing specific cognitive functions. NR generates highly immersive and interactive environments that realistically simulate real or fictitious situations, involving multiple sensations and movements. This can increase involvement and the potential effectiveness of these methods. Therefore, the effectiveness of these interventions should be analyzed separately or in comparison with traditional methodologies, as conclusions about NR could be compromised due to differences in these aspects (Rizzo and Koenig, 2017).

Other exclusion criteria included opinion articles, literature reviews, articles restricted to other cognitive deficits excluding executive deficits, and studies focusing solely on substance use disorder without addressing alcohol.

### 2.2 Data selection and extraction

After the initial screening of titles and abstracts, two researchers analyzed the full articles independently according to the defined inclusion and exclusion criteria. Any disagreements were resolved with a third researcher. One researcher extracted the data into a standardized table to systematize the information, and the other researcher verified it. The complete studies that were selected were subsequently analyzed. This analysis included the information defined in the systematic literature review protocol (Ferreira et al., 2024). The data were aggregated into three tables corresponding to the study characterization, including author, year of publication, study design, inclusion and exclusion criteria, and population and participant characterization, such as number, age, sex, comorbidity, consumption type, and other consumption. The tables also included NR program objective, tasks, strategies, cognitive domains trained, intervention context, number of rehabilitation sessions/h, concomitant interventions, control group, transfer effect, follow-up, outcome measure (participation rate), main outcome (changes in cognitive domains), and other outcomes (alcohol consumption, emotional aspects, and functionality in daily life). A narrative analysis of the results and an assessment of the risk of bias were carried out.

### 2.3 Risk assessment of bias

Two researchers independently evaluated the methodological quality of the selected studies using the same distribution methodology. Any disagreements were resolved by a third researcher. The risk of bias was evaluated using the Cochrane Collaboration tool for assessing risk in randomized trials (RoB 2) (Sterne et al., 2019). This tool evaluates six domains and classifies the risk of bias as critical, serious, moderate, low, or insufficient. Evaluating each domain enables one to determine the general risk of bias for the result under evaluation. When information was missing, the authors were contacted to clarify aspects and obtain information regarding absent methodological procedures. No response was received.

### 2.4 Assessment of the certainty of the evidence

The overall certainty of the evidence was assessed using the GRADE methodology (Guyatt et al., 2008), which analyzes the risk domains of bias, inconsistency, indirectness, imprecision, and publication bias. This approach enabled us to evaluate the strength of the conclusions and pinpoint significant methodological limitations in the included studies.

### 2.5 Data synthesis

Data synthesis was performed to describe the sample, study design, and inclusion and exclusion criteria. The various NR programs studied were analyzed based on the variables that characterize them, as described in the article. The instruments and evaluation points (baseline and follow-up) were analyzed. The main results of the various NR studies regarding cognitive domains were reported, as well as other results such as changes in consumption, emotional aspects, and functionality in daily life.

Due to the heterogeneity of the included studies and NR programs, a meta-analysis was not possible. Therefore, we carried out a structured narrative synthesis was carried out, following the SWiM (Synthesis Without Meta-analysis) guidelines (Campbell et al., 2020), grouping the studies according to the relevant characteristics of the interventions and outcomes. This allowed us, in order to identify patterns and facilitate the standardization of NR programs and studies in this area. The direction of the effect, the consistency of the findings, and influence of potential confounding factors were systematically analyzed, as were the strengths, weaknesses, and gaps in the evidence.

## 3 Results

**Figure 1:**
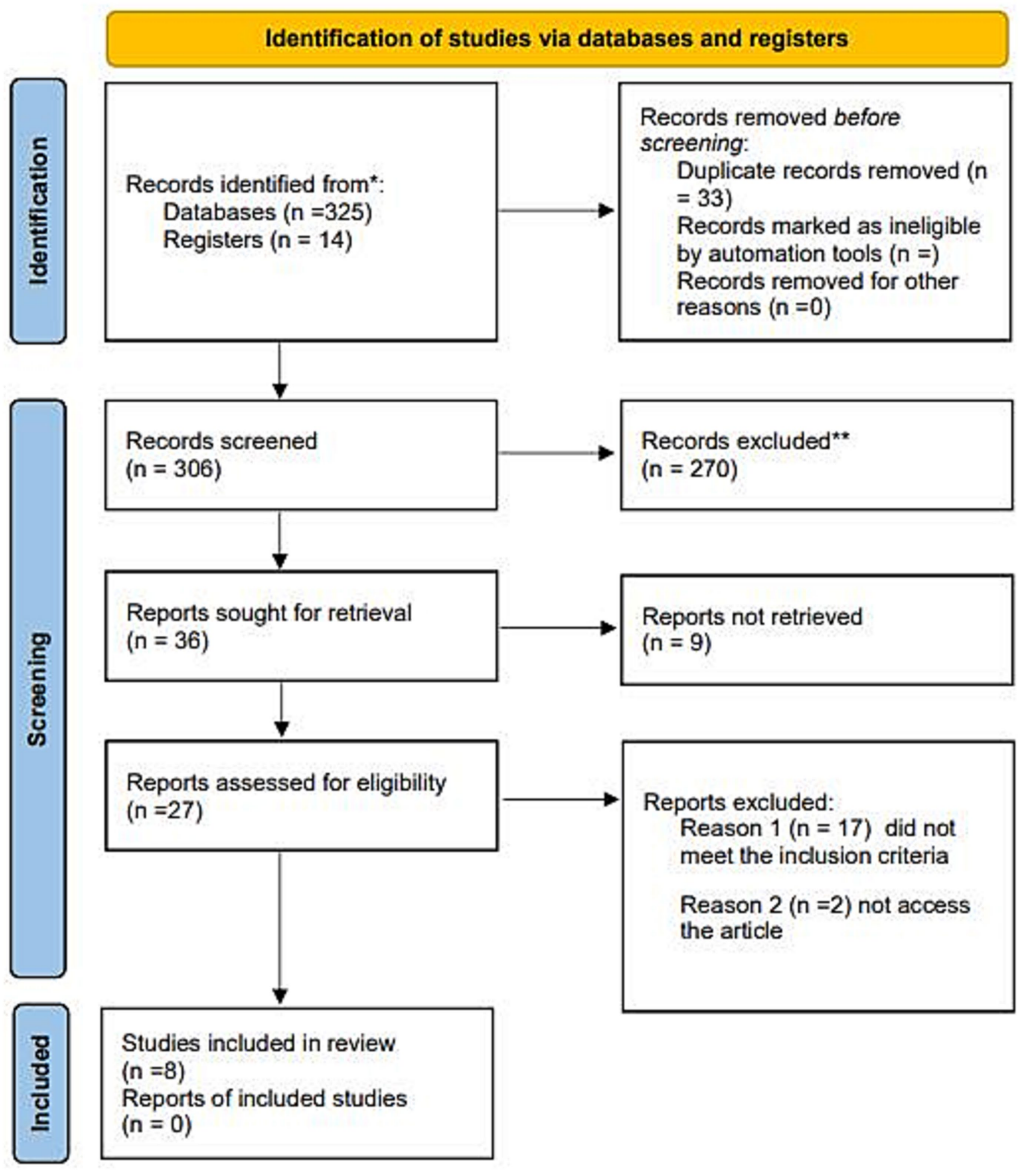
PRISMA flow diagram for study selection.

Of the initial 339 articles, 306 were selected for a preliminary analysis based on their titles and abstracts, after eliminating duplicates (Figure 1). Of these, 27 articles were deemed eligible (see Supplementary material 1 for reasons articles were excluded), but only eight were chosen for the final analysis.

### 3.1 Characteristics of the studies

The eight randomized clinical trials examined the impact of NR on cognitive functioning in AUD treatment programs in hospital units or therapeutic communities (Table 3; Supplementary material 2). Some of these trials also analyzed the association between NR and alcohol consumption-related variables (Rupp et al., 2012; Mathai et al., 1998; Wanmaker et al., 2018; Kumar et al., 2019).

**Table 3:** Characteristics of the studies included in the systematic literature review.

Author, year	*N*	Age	Sex	Co-morbidity	Other consumption
Rupp et al. (2012)	37	Up to 65 years old; experimental group—mean age 45.2 years (SD = 10.5); control group—mean age 45.5 years (SD = 8.8)	Both	Mental disorders; and neurological diseases—excluded	Excluded (only included cannabis consumption)
Kumar et al. (2019)	50	18–45 years old; experimental group—mean age 34.28 years (SD = 5.33); control group—mean age 34.08 years (SD = 5.73)	Both	Excluded	Not reported
Gamito et al. (2013)	61	Mean age 45.64 years (SD = 9.67)	Both	Not reported	Heroin abuse
Gamito et al. (2014b)	42	Mean age 45.45 years (SD = 10.31); control group—mean age 48.61 years (SD = 8.02); experimental group—mean age 41.62 years (SD = 11.4)	Both	History of previous neurological diseases—excluded	Excluded
Gamito et al. (2014a)	54	Mean age 45.37 years (45.37 SD = 10.12)	Both	History of previous neurological diseases—excluded	Excluded
Mathai et al. (1998)	8	30–45 years old	Male	Not reported	Excluded
Wanmaker et al. (2018)	74	16–70 years old; experimental group—mean age 38.53 years; control group—mean age 36.6 years	Both	Psychotic episode (last month); severe brain damage; and neurodegenerative diseases—excluded	Cocaine and cannabis use disorder
Snider et al. (2018)	31	18–65 years old; experimental group—mean age 42.5 years (SD = 2.0); control group—mean age 42.4 years (SD = 2.3)	Both	History of stroke; seizures; and loss of consciousness—excluded	Not reported

*N*, number of participants at the end of the investigation; SD, standard deviation.

The average sample size was 50 participants (standard deviation [SD] = 19.5; median = 50), ranging from a minimum of eight participants (Mathai et al., 1998) to a maximum of 74 participants (Wanmaker et al., 2018). Almost all of the trials included both genders, except for one trial that only involved males (Kumar et al., 2019).

Despite excluding individuals under 18 or over 65 years old, we included a study with a sample ranging in age from 16 to 70 years old (Wanmaker et al., 2018), in which early adulthood participants were compared to adults over 24 years old. We included this study because it examined situations of dependence in both groups, aligning with the framework of this review. Furthermore, the study was not limited to the older age group (average age of the experimental group = 38.53 years; average age of the control group = 36.6 years). Thus, the age range of the sample was 16–70 years old, with an average age of 42 years (SD = 4.1; median = 43).

In this area of research, the inclusion criteria appear to be very broad. Common selection criteria include a diagnosis of alcohol dependence (present in all clinical trials), a specific age range (Rupp et al., 2012; Mathai et al., 1998; Kumar et al., 2019), cognitive performance (Rupp et al., 2012; Mathai et al., 1998; Gamito et al., 2014b), and abstinence (Mathai et al., 1998).

Psychiatric comorbidity and neurological problems, including severe brain damage, a history of stroke or seizures, and loss of consciousness, were excluded (Gamito et al., 2014a,b; Rupp et al., 2012; Snider et al., 2018; Wanmaker et al., 2018; Kumar et al., 2019). The careful control of these variables in the studies is a positive aspect, as it could bias the analysis of cognitive recovery with NR.

Regarding other substance use disorders, two studies included cases of heroin, cocaine, and cannabis dependence (Wanmaker et al., 2018; Kumar et al., 2019), and one excluded polydrug use but included cannabis (Rupp et al., 2012). Furthermore, two studies did not provide information on this (Snider et al., 2018; Kumari and Kumar, 2020; Gamito et al., 2014b).

Four of the clinical trials had dropouts during the study (Gamito et al., 2014a,b; Rupp et al., 2012; Snider et al., 2018). The reasons for dropping out were not fully meeting the inclusion criteria (Rupp et al., 2012; Snider et al., 2018), unknown reasons (Rupp et al., 2012; Gamito et al., 2014b), alcohol consumption (Rupp et al., 2012), change of address (Gamito et al., 2014b), time elapsed between sessions (Snider et al., 2018), and refusal or voluntary removal from the study (Gamito et al., 2014a; Snider et al., 2018).

### 3.2 Cognitive rehabilitation programs

Half of the clinical trials primarily aimed to assess the program’s impact on cognitive functioning (Gamito et al., 2013, 2014a,b; Snider et al., 2018). The other half also evaluated the program’s effects on clinical variables, such as alcohol consumption and wellbeing/quality of life (Rupp et al., 2012; Mathai et al., 1998; Wanmaker et al., 2018; Kumar et al., 2019) (Table 4; Supplementary material 3).

**Table 4:** Characterization of included neuropsychological rehabilitation programs.

Author, year	Type	Executive domains available	Strategies of intervention	Number of sessions/duration (days, hours)
Rupp et al. (2012)	Multidomains	Working memory; and inhibition	Technology	12 sessions/4 weeks/3 sessions per week (45–60 min per session)
Kumar et al. (2019)	Multidomains	Mental flexibility; working memory; and inhibition	Not reported	Number of sessions—not reported/18 days;
Gamito et al. (2013)	Multidomains	Working memory; planning; and cognitive flexibility	Technology	8–12 sessions/1 month/2–3 sessions per week (starting from the sixth day of treatment)
Gamito et al. (2014b)	Multidomains	Working memory	Technology/paper-and-pencil	10 sessions/4 weeks/3 sessions per week (50–60 min per session)
Gamito et al. (2014a)	Multidomains	Working memory; and planning	Technology	10 sessions/4 weeks/2–3 sessions per week
Mathai et al. (1998)	Multidomains	Planning	Technology/paper-and-pencil	43 sessions/6 weeks/1 session per day
Wanmaker et al. (2018)	One domain	Working memory	Technology	24 sessions/1 week (25 min per session)
Snider et al. (2018)	One domain	Working memory (verbal and visuospatial)	Technology	20 sessions/session duration not reported

Number of sessions, number of session; duration of rehabilitation; session duration.

Most of these trials aimed to address several components of EFs simultaneously (Gamito et al., 2013, 2014a,b; Rupp et al., 2012; Mathai et al., 1998; Kumar et al., 2019), while only two focused on a single domain (Snider et al., 2018; Wanmaker et al., 2018). The EFs covered included working memory (Gamito et al., 2013, 2014a,b; Rupp et al., 2012; Snider et al., 2018; Stavro et al., 2013; Kumar et al., 2019), planning (Gamito et al., 2013, 2014a; Mathai et al., 1998), inhibition (Rupp et al., 2012; Kumar et al., 2019), and flexibility (Kumar et al., 2019; Gamito et al., 2013).

Five of the analyzed NR programs used technological resources (Rupp et al., 2012; Snider et al., 2018; Wanmaker et al., 2018; Gamito et al., 2013, 2014b), and two used a combination of technological resources and pen-and-paper exercises (Gamito et al., 2014a; Mathai et al., 1998).

Most of the NR programs were used alongside standard AUD treatments (Gamito et al., 2013, 2014a; Rupp et al., 2012; Mathai et al., 1998; Wanmaker et al., 2018; Kumar et al., 2019). The analysis revealed heterogeneity in the duration of the NR programs (from one to 6 weeks), with most lasting approximately 1 month (Gamito et al., 2013, 2014a,b; Rupp et al., 2012). The same was true for the number of sessions. Some programs had fewer sessions, ranging from eight to 12 (Gamito et al., 2013, 2014a,b; Rupp et al., 2012), and others had more sessions, ranging from 20 to 43 (Snider et al., 2018; Wanmaker et al., 2018).

In most trials, the control group only received treatment as usual. In two trials, the NR was used with characteristics different from those of the experimental group. For example, the difficulty level of the tasks differed, or a neutral stimulus was used (Snider et al., 2018; Wanmaker et al., 2018).

Almost all trials described a variety of cognitive stimulation tasks. One example is the online working memory training program (Cogmed) (Snider et al., 2018). Another example is the dual n-back task (Wanmaker et al., 2018). This type of task involves indicating when a stimulus from one or more modalities is equal to stimulus n from previous steps. Two of these trials also included relaxation (Rupp et al., 2012; Kumar et al., 2019).

### 3.3 Measures and findings of outcomes

Some trials analyzed the transfer effect (Rupp et al., 2012; Snider et al., 2018; Mathai et al., 1998; Wanmaker et al., 2018; Kumar et al., 2019) (Table 5; Supplementary material 4). One study found a transfer effect in other cognitive domains (Wanmaker et al., 2018) as well as in non-cognitive domains such as substance use substances and psychological wellbeing (Rupp et al., 2012). Another study found a transfer effect in relapse prevention and prolonged abstinence from alcohol consumption (Kumar et al., 2019). However, others did not find a transfer effect from NR to family functioning or long-term abstinence (Snider et al., 2018; Mathai et al., 1998).

**Table 5:** Results of neuropsychological rehabilitation.

Author, year	Evaluation moments	Cognitive outcomes/cognitive results	Transfer effects
Rupp et al. (2012)	Baseline: beginning of inpatient treatment; follow-up: after NR/at the end of treatment (4 weeks)	Improvements with neuropsychological rehabilitation: working memory; and no improvements in cognitive inhibition	Transfer effects: non-cognitive outcomes and untrained cognitive domains
Kumar et al. (2019)	Baseline assessment (after three to four days of detoxification); follow-up: after 18 days of treatment (followed up for 6 months to compare abstinence)	Improvements with neuropsychological rehabilitation: domains of executive functions; and affect regulation	Transfer effects: relapse and long-term abstinence
Gamito et al. (2013)	Baseline assessment (before NR); follow-up: 4 weeks (at least 30 days between evaluations)	Improvements with neuropsychological rehabilitation: general cognitive abilities; and executive functioning	Not reported
Gamito et al. (2014b)	Baseline assessment (before NR/first and fourth day after the first evaluation); follow-up: after 30 days	Improvements with neuropsychological rehabilitation: executive functions	Not reported
Gamito et al. (2014a)	Baseline assessment—after screening; follow-up: at least 30 days later	Improvements with neuropsychological rehabilitation: flexibility and general cognitive abilities (psychomotor processing speed, and attentional ability) in both groups. However, there were more improvements in executive functions in the experimental group	Not reported
Mathai et al. (1998)	Baseline assessment; follow-up: 6 weeks	Improvements with neuropsychological rehabilitation: information processing; and memory	Transfer effects: speed of serial information processing; memory No transfer effects: family functioning; and long-term abstinence
Wanmaker et al. (2018)	Baseline assessment; follow-up: 1 week	Improvements with neuropsychological rehabilitation: working memory (both groups showed better scores on the trained tasks; however, the control group showed more improvements); and improvements in the 3 components were only seen in the INTENSIVE group	No transfer effects: craving, substance abuse, impulsivity, and psychopathology
Snider et al. (2018)	Baseline assessment; follow-up: after NR	Improvements with neuropsychological rehabilitation: performance on near-transfer task (experimental group)	No transfer effects: family functioning; and long-term abstinence

NR, Neuropsychological Rehabilitation.

All the studies included an evaluation before and after the NR intervention. The latter evaluation depended on the intervention’s duration. The most frequent evaluation occurred after 1 month (Gamito et al., 2013, 2014a,b; Wanmaker et al., 2018).

All clinical trials used a series of neuropsychological tests, except for one trial that used only one neuropsychological test and two screening tests for cognitive deficits (Gamito et al., 2014a).

Analysis of the cognitive outcomes revealed that all trials demonstrated improvement in the examined EFs, except for one trial in which improvement was observed in only one of the analyzed components (working memory) and not the other (inhibition) (Rupp et al., 2012).

### 3.4 Risk of bias

**Figure 2:**
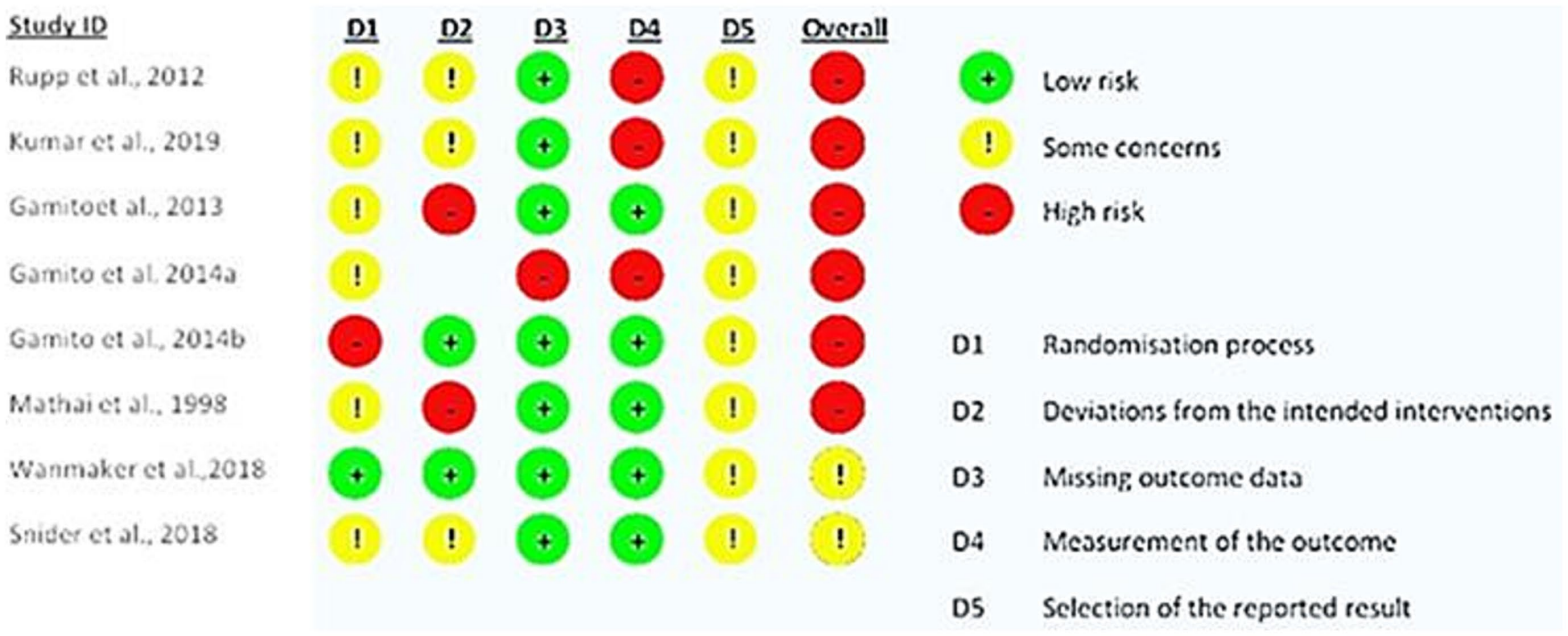
Results of the risk of bias assessment in randomized trials (RoB 2).

We identified six studies with a high risk of bias (Gamito et al., 2013, 2014a,b; Rupp et al., 2012; Mathai et al., 1998; Kumar et al., 2019) and two studies with restrictions (Snider et al., 2018; Wanmaker et al., 2018) (Figure 2). Problems related to methodology and a lack of essential information were identified, including randomization, double blinding, initial planning, intended interventions, and data presentation. The risk of bias when measuring results is significant (Gamito et al., 2014a; Rupp et al., 2012; Kumar et al., 2019). There is no evidence that researchers or participants were unaware of the allocated group (Rupp et al., 2012; Gamito et al., 2013). There is no mention of changes to the proposed intervention or missing data due to participants lost to follow-up (Gamito et al., 2014b). Restrictions include issues with the randomization process (Snider et al., 2018) and the selection of reported results (Snider et al., 2018; Wanmaker et al., 2018). This investigation suggests that future studies should increase the rigor of their methodologies, particularly with regard to the randomization of samples, and standardize procedures and variables concerning NR in the population.

After analyzing both articles with restrictions, we found that the results did not change significantly, except for a minor reduction in the average age and less dispersion in certain variables, such as sample size, number of NR sessions, type of intervention in the control group, and tests used. The NR programs focused on a single domain.

### 3.5 Certainty of evidence

The certainty of the evidence was assessed using the GRADE (Guyatt et al., 2008) methodology (Table 6). It was considered low mainly due to the risk of serious bias and inconsistency in the results due to heterogeneity. The populations and interventions were adequate; however, the evidence revealed imprecision due to small samples and weak estimates. No indicators of publication bias were detected, though they cannot be completely excluded.

**Table 6:** Analysis of the certainty of the evidence.

Domain	Assessment	Explanation
Risk bias	Very serious (2-level reduction)	6 out of 8 studies showed a high risk
Inconsistency	Serious (1-level reduction)	Variability between studies
Indirectness	Not serious	Adequate population and interventions
Imprecision	Serious (1 level reduction)	Small samples and imprecise estimates
Publication Bias	Not serious	Not identified, but possible

### 3.6 Narrative and analytical synthesis of the results

Due to the heterogeneity of the included studies at the level of intervention protocols, program duration, assessment instruments, sample characteristics, and outcomes, a meta-analysis could not be performed. Therefore, a narrative and critical analysis of the results was carried out based on SWIM guidelines (Campbell et al., 2020). The studies were grouped according to their relevant characteristics and interventions to establish patterns.

#### 3.6.1 Effect direction

All studies reported improvements in at least one component of the studied executive functions after neurorehabilitation. Seven studies reported consistently positive effects. One study found an improvement in WM without an effect on inhibition, showing partial benefits of NR (Rupp et al., 2012). No study reported cognitive deterioration.

#### 3.6.2 Comparison according to number of sessions

The number of sessions ranged from eight to 43. The comparative analysis did not reveal a clear pattern between the number of sessions and the magnitude of improvement. Short/moderate-duration programs (8–12 sessions) (Gamito et al., 2013, 2014a,b; Rupp et al., 2012) and long-duration programs (20–43 sessions) (Snider et al., 2018; Mathai et al., 1998; Wanmaker et al., 2018) showed similar improvements. Very intensive programs (e.g., 24 sessions in 1 week) (Wanmaker et al., 2018) also did not demonstrate superior effects compared to the others. Based on these data, the number of sessions does not appear to be a determining factor for the observed effect.

#### 3.6.3 Comparison according to the number of domains intervened

Six studies used multi-domain programs (Rupp et al., 2012; Mathai et al., 1998; Kumar et al., 2019; Gamito et al., 2013, 2014b) and two focused on a single domain (WM) (Snider et al., 2018; Wanmaker et al., 2018). The multi-domain programs produced more extensive improvements and were the only ones to demonstrate transfer effects to other cognitive domains or clinical variables, such as consumption, abstinence, and wellbeing) (Rupp et al., 2012; Kumar et al., 2019). Therefore, multidomain-centered neural research appears to promote generalization of the effects to untrained cognitive domains and clinical variables.

#### 3.6.4 Impact on non-intervention domains (transfer effect)

Five studies evaluated the transfer effect (Rupp et al., 2012; Snider et al., 2018; Mathai et al., 1998; Kumar et al., 2019; Wanmaker et al., 2018). Three of these studies demonstrated this effect for other untrained cognitive domains or clinical variables, such as compulsion, wellbeing, and prolonged abstinence) (Rupp et al., 2012; Mathai et al., 1998; Kumar et al., 2019). It is worth noting that the studies that found a transfer effect were the only RN programs that included multiple domains.

#### 3.6.5 Comparison according to the type of tests used

Tests that screen for global cognitive functioning (e.g., MoCA, MMSE) capture broader improvements. More specific tests (e.g., n-back, Stroop, TMT) detected improvements restricted to the trained components. One study that used only one test presented less robust results. Based on these data, we can infer that the sensitivity of the instruments influences the detection of effects.

#### 3.6.6 Comparison according to evaluation time points

Most studies used a 1-month follow-up, while two studies included an extended follow-up of 6 months. No major differences were found, though the studies with 6-month follow-up showed partial maintenance of results (Mathai et al., 1998). The absence of a longer follow-up period may limit our understanding of the durability of the effects of NR programs.

#### 3.6.7 Comparison with traditional treatment

Most trials used traditional treatment for AUD as a passive control without including any form of equivalent cognitive training. This limits the ability to isolate the specific effects of the intervention (Gamito et al., 2013, 2014a; Rupp et al., 2012; Mathai et al., 1998; Kumar et al., 2019). Two studies included active controls, enabling a more rigorous comparison between NR and alternative cognitive tasks (Snider et al., 2018; Wanmaker et al., 2018). The heterogeneity in the definition of the comparator may constrain the interpretation of the results since the improvements found in the experimental groups may be associated with not only with the effect of NR but also with the spontaneous cognitive recovery that occurs during the first weeks of abstinence.

#### 3.6.8 Comparison of alcohol consumption with polydrinking

Consistent and positive cognitive improvements are found in the group with exclusive alcohol consumption compared to the group with polyconsumptions (Gamito et al., 2014a; Wanmaker et al., 2018). Concomitant consumption can enhance the neurotoxic effects of each substance, leading to greater cognitive impairment and longer recovery times (Jonides, 2004).

#### 3.6.9 Technology integration in NR

The integration of technology appears promising in NR in AUD. Studies that included only technology, compared to those that combine it with pencil-and-paper exercises (Mathai et al., 1998; Gamito et al., 2014b), seem to have a greater expression of the effect studied.

#### 3.6.10 Integration of risk of bias into the synthesis

Six studies presented a high risk of bias (Gamito et al., 2013, 2014a,b; Rupp et al., 2012; Mathai et al., 1998; Kumar et al., 2019), and two revealed some concerns at this level (Snider et al., 2018; Wanmaker et al., 2018). Studies with the lowest risk of bias did not show substantially different results, suggesting consistency in the direction of the effect. However, the certainty of the evidence is limited.

Based on these patterns, the following aspects can be identified, based on SWIM analysis (Campbell et al., 2020):

##### 3.6.10.1 Strengths

Studies report improvements in the components of executive function studied with NR. The effectiveness of NR seems to depend more on the diversity of tasks used to intervene in multiple domains than on the duration and number of sessions in the programs. Programs with multidomain integration, on the other hand, show a greater transfer effect. Using standardized assessment measures adds value by providing greater robustness to the data.

##### 3.6.10.2 Weaknesses

The control groups are predominantly passive (usual treatment), which does not allow for the isolation of the specific effects of NR. Some studies have small sample sizes and experience losses during the study. The heterogeneity of programs, samples, and instruments can limit comparability between studies. The high risk of bias reduces the overall certainty of the evidence.

##### 3.6.10.3 Insight

The effectiveness of NR depends essentially on integrating multiple EFs components and diverse tasks, or complex interventions, rather than on program duration and intensity. These reveal broader effects with greater transfer to untrained domains or clinical variables. An exclusive focus on a single domain allows for improvement in that domain, but does not produce generalizations to untrained or clinical variables. This data suggests that interventions restricted to a single cognitive domain may be insufficient to produce relevant behavioral changes in the context of addiction. The duration of the programs is not clearly associated with the magnitude of the effect. The sensitivity of the instruments determines whether this effect is detected.

##### 3.6.10.4 Missing pieces

The absence of active control groups limits our ability to distinguish the specific effects of NR, and the lack of follow-up beyond 6 months prevents us from drawing solid conclusions about the durability of the effects achieved with NR. There is a lack of a standardized NR model for AUD which could serve as a reference for interventions with this population.

## 4 Discussion

### 4.1 The main finding of the study

The main objective of this review is to evaluate the effectiveness of NR in improving EFs in AUD. The results from the eight included clinical trials suggest that RN has the potential to improve the components of the studied EFs in AUD patients (Rupp et al., 2012; Mathai et al., 1998; Wanmaker et al., 2018; Gamito et al., 2013). This differs from previous reviews, which highlighted incongruity in the results (Caetano et al., 2021). Improvements were found in all studied components of EFs studied, such as planning, inhibition, flexibility and WM. WM is the most integrated component, either alone or with other components simultaneously. This may be associated with the fact that WM is one of the components most affected by alcohol consumption and strongly correlates with treatment success (Caetano et al., 2021). These results suggest that NR may be a valuable addition to traditional AUD treatments, promoting cognitive and environmental recovery and improving clinical outcomes.

In this context, although the effect of NR (performance improvement) was consistent, its magnitude varied across studies. This variation appears to be associated with the heterogeneity of the programs, samples and instruments used. Some relevant patterns were identified that may facilitate the standardization of these programs. For example, NR is more effective when it focuses on multiple EF components simultaneously (Hofmmann et al., 2012). Additionally, these effects can be transferred to clinical variables, such as long-term abstinence and wellbeing (Rupp et al., 2012). This effect involves both trained and untrained tasks that require the same cognitive skills and share neuroanatomical areas and circuits (Roebuck-Spencer et al., 2017). The benefits of NR can be generalized to other functions, tasks, or contexts that were not directly addressed. This is important when discussing AUD, especially in terms of recovery, as different EFs are affected by consumption, relapse prevention and reintegration into daily life after treatment (Hofmmann et al., 2012). This promotes a more comprehensive and targeted approach to recovery for these individuals. Conversely, as the results show, acting on different components amplifies the transfer effect, particularly in other cognitive or life areas. Many studies have already integrated this analysis, demonstrating a shift in focus from solely studying the recovery of cognitive domains to studying these programs and generalizing them to consumption, to facilitate alcohol rehabilitation. This analysis has not been highlighted in previous reviews (Caetano et al., 2021).

Overall, the results of this review partially align with those of previous reviews and primary studies. Improvements in EFs were also identified after cognitive training or NR interventions in AUD. However, some reviews reported greater inconsistency in the results, which may be due to the inclusion of studies with more heterogeneous methodologies or less structured interventions (Caetano et al., 2021).

This review contributes to the literature by critically analyzing the structure of NR programs, including the duration and number of sessions, the domains trained, and the tests used. This analysis clarifies these elements and facilitates the standardization of NR in AUD.

Various terms are often used for executive function recovery programs, and some studies have even emphasized the difficulty of classifying them (Gamito et al., 2014a,b). In this review, however, most studies classified their programs as cognitive rehabilitation, cognitive training, or cognitive stimulation; only one study defined its program as cognitive rehabilitation.

The duration of NR programs is often less than 1 month, and no differences in effectiveness were found based on the number of sessions. However, this data is inconsistent with the literature, which suggests that a longer recovery time is necessary to address the significant and habitual impact on executive functioning in this population, as well as to reorganize its neural networks in order to compensate for or restore deficits in the various components of EFs. It is important to note that many of the included studies involved participants in the early stages of abstinence; the improvements may be associated with the effect of cessation of consumption rather than the effects of NR (Mann et al., 1999). Other neuropsychiatric pathologies establish guidelines of three to 6 months for mild to moderate deficits and 1 year for severe deficits (Mutcnick, 1988). This reinforces the need to increase the duration of NR programs. Learning new strategies and recovering or reinforcing EFs occurs over the course of several sessions. This requires sufficient time for these skills to be practiced, consolidated, and generalized to daily life (Cicerone et al., 2000). Furthermore, literature highlighting that residual damage may persist for years after abstinence underscores the fragility of these programs. Other reviews demonstrate variation in the number of sessions and NR duration without critically addressing this issue (Caetano et al., 2021). Additionally, the literature describes critical recovery periods, such as 1 month, 3 months, and 1 year. Often, these periods are associated with an inability to develop the necessary skills and strategies to cope with the different challenges of these stages.

The absence of equivalent active control groups and prolonged follow-up limits our ability to assess the effectiveness of these programs. These arguments underscore the importance of carefully stabilizing abstinence before the intervention begins and of integrating longer periods so as not to be influenced by spontaneous recovery (Stavro et al., 2013).

The effectiveness of NR is influenced by the neuropsychological test battery used; greater sensitivity of neurospychological instruments may lead to greater detection of improvements. Several measures should be included, such as validated neuropsychological tests with psychometric properties and screening tests. This approach considers variables that could affect performance, such as alcohol consumption and polyconsumption, allowing for a more precise and robust assessment (Roebuck-Spencer et al., 2017). Standardizing a battery of tests to assess EFs in AUD seems difficult. However, certain neuropsychological tests are commonly used, such as the stroop test, the digit span test, the trail making test, and the restriction test (FAB Supplementary material 3). These tests are widely used in clinical practice and have well-studied psychometric characteristics. Previous reviews (Caetano et al., 2021) have also described this preference for these tests, which may serve as a reference when selecting measures for studies in this area.

The methodologies used for this purpose have evolved significantly with the adoption of technology, such as computerized or mobile training. However, they still maintain the principles and guidelines of traditional NR. The efficacy of these methods has been demonstrated (Gamito et al., 2013, 2014a,b). Previous reviews have also noted this trend, though they found a higher prevalence of pencil-and-paper exercises (Caetano et al., 2021). The use of technology seems to add value by motivating participants (Mutcnick, 1988), encouraging adherence to treatment, facilitating learning, and adapting the complexity of exercises to the level of need or severity of the deficit.

### 4.2 What the study adds

This review focuses on AUD and the analysis of EFs, making it innovative and extremely relevant. These functions are important for this population due to their prevalence and impact on alcohol recovery. Focusing on EFs rather than general clinical results is important because improving these functions is essential for the effectiveness of standard AUD treatments. This has not yet been explored in previous reviews (Caetano et al., 2021). For example, improved EFs can lead to better impulse control and planning, which can prevent relapses and improve quality of life (Rupp et al., 2012; Mathai et al., 1998).

This review emphasizes the importance of standardizing these programs while considering the unique needs of this population and the factors that promote recovery. Therefore, studies must pay special attention to the characteristics that define these programs. For example, they should consider how evaluation is conducted, which strategies and methodologies should be used, and how frequently the programs should be conducted to be effective. This review seeks to overcome gaps in previous reviews (Caetano et al., 2021) by analyzing these structural variables, particularly the total duration of the programs, in more detail.

Despite their limitations, the results have several implications for clinical practice and the planning of future studies, allowing us to establish guidelines:

- The potential effect of NR on the recovery of FEs demonstrates that NR can complement traditional AUD treatments. This highlights the need to regularly integrate NR into services operating in this area to promote comprehensive cognitive and alcohol rehabilitation interventions (Bates et al., 2023; Rupp et al., 2012);
- The evidence showing that NR programs integrating different EFs components simultaneously produce broader effects and a greater probability of transferring to clinical variables serves as a basis for structuring them. The evaluation of NR effectiveness should incorporate an assessment of the transfer effect (Fals-Stewart and Lam, 2010);
- NR programs should also be based on a comprehensive, multidimensional assessment that includes the various components of EFs. This assessment should use robust, validated neuropsychological tests with psychometric qualities. It should also consider variables that may influence recovery, such as comorbidities, alcohol use, and substance use. Based on this review, some neuropsychological tests appear to be consensus-based for assessing these components, consistent with other reviews (Caetano et al., 2021). This facilitates the standardization of assessment protocols. More global tests tend to capture broader improvements, while more specific tests target the trained components. It may be necessary to integrate both types of tests into the protocols;
- The duration of NR is a variable that requires further clarification. Although it was not a determining factor in the studied effect, it is important to note that improvement in the first month may be linked to spontaneous recovery and initial abstinence. It may not represent the actual effect of NR (Stavro et al., 2013). It is necessary to monitor the evolution over a longer period that is less sensitive abstinence’s effect;
- The integration of technology into these programs appears advantageous, given its demonstrated effectiveness in making these processes more dynamic and motivating. This promotes adherence and the ability to adjust the level of difficulty of training according to individual needs (Mutcnick, 1988);
- The importance of an assessment before and after NR is emphasized as a means of ensuring progress monitoring. Prolonged follow-up is necessary to evaluate the durability of the effects (Stavro et al., 2013).

### 4.3 Limitations of this study

The diversity of methodologies within the scope of NR programs (e.g., session duration, frequency, and number; intervention type; measures; and evaluation times) hinders the evaluation of their effectiveness in AUD conditions. This makes comparisons between studies difficult and renders meta-analysis impossible. A narrative approach was used to synthesize the data, assess the risk to life, and determine evidence certainty. Additionally, a meta-analysis could not be carried out due to the small sample size and studies with a significant risk of bias.

Although there are indications of improvements in FEs with NR in AUD, it is important to emphasize that the findings of this review do not allow us to conclude on its effectiveness. Most of the included studies have methodological limitations that prevent robust causal inferences. These limitations include the absence of equivalent active control groups, heterogeneous interventions, inclusion of participants in the early stages of abstinence, short follow-up periods, small sample sizes (Mathai et al., 1998), dropout rates (Snider et al., 2018; Gamito et al., 2014b), high risk of bias (Gamito et al., 2013, 2014a,b; Rupp et al., 2012; Mathai et al., 1998; Kumar et al., 2019) and low certainty of evidence (Guyatt et al., 2008). Therefore, the results should be interpreted as descriptive and exploratory, reflecting patterns observed in the integrated studies. These results can facilitate the establishment of guidelines for future studies and clinical practice.

One limitation of this review is that strict inclusion criteria were established, which limited the sample to clinical trials and excluded all other types of studies. However, this approach helped control certain variables and standardize the studies, allowing for more precise conclusions to be drawn and facilitating reflection on this topic.

Additionally, the failure of the studies to differentiate between the severity of AUD is considered a limitation because more severe cases may result in more significant damage and a different recovery process. Future studies should compare results based on AUD severity (Bates et al., 2023).

Another limitation is that the analysis included different interventions (e.g., cognitive training, cognitive remediation, and NR), and it was not possible to group the interventions for comparison due to the small sample size.

Given these limitations, future studies must employ more rigorous methodologies, use larger sample sizes, and standardize the duration, number of sessions, and structure of these programs. Additionally, the results multidomain programs must be replicated to confirm their superior efficacy, incorporating follow-up periods of at least 6 months. Future research should also explore the relationship between improvements in executive functions and relevant clinical outcomes, such as relapse, treatment adherence, and daily functioning.

## 5 Conclusion

Further rigorous studies on the effectiveness of NR in PUA are necessary, as well as the standardization of the variables that characterize these programs. This will facilitate research in this area and enable reliable comparisons to support clinical practice. It is important to corroborate the implementation of these programs with knowledge obtained from studies to highlight their advantages and integrate them more regularly as adjunct treatments to traditional treatments. Therefore, guidelines must be established to prevent these programs from merely replicating those used for other pathologies, as this population has specific needs in the different phases of treatment (Le Berre et al., 2017; Bates et al., 2023). It is worth noting that, although suggests that these programs are beneficial.

## Data Availability

The original contributions presented in the study are included in the article/Supplementary material, further inquiries can be directed to the corresponding author.
