# Supplementary material for: Neuropsychological rehabilitation in executive deficits resulting from alcohol use disorder: systematic review of literature
Source: Front Psychol. 2026 Apr 29;17:1805577. doi: 10.3389/fpsyg.2026.1805577 (PMC13168111; doi:10.3389/fpsyg.2026.1805577)
Supplement: Supplementary file 1 [file Table_1.DOCX]

**Supplementary Material 1: Reasons for excluding the articles**

| **Authors** | **Title** | **Reason for exclusion** |
| --- | --- | --- |
| Jones et al., 2018^101^ | A Randomized Controlled Trial of Inhibitory Control Training for the Reduction of Alcohol Consumption in Problem Drinkers | Context/ sample |
| Berry et al., 2019^102^ | A stepped wedge cluster randomized trial of a cognitive remediation intervention in alcohol and other drug (AOD) residential treatment services | Severe psychiatric and neurological comorbidity |
| Eberl et al., 2013^103^ | Approach bias modification in alcohol dependence: Do clinical effects replicate and for whom does it work best? | Missing main outcome |
| Marceau et al., 2017^104^ | Cognitive remediation improves executive functions, self-regulation and quality of life in residents of a substance use disorder therapeutic community | Severa neurological comorbidity |
| Bell et al., 2016^105^ | Cognitive Training and Work Therapy for the Treatment of Verbal Learning and Memory Deficits in Veterans With Alcohol Use Disorders | Context |
| Rupp et al., 2012^82^ | Cognitive remediation therapy during treatment for alcohol dependence. | Included |
| Gamito et al., 2014b^88^ | cognitive stimulation through mHealth-based program for patients with alcohol dependence syndrome: A randomized controlled study | Included |
| Bickel, 2014^106^ | Computerized Working-Memory Training as a Candidate Adjunctive Treatment for Addiction | Review |
| Alfonso et al., 2011^107^ | Combined goal management training and mindfulness meditation improve executive functions and decision-making performance in abstinent polysubstance abusers | Context |
| Kumar et al., 2019^84^ | Effectiveness of an Integrated Intervention Program for Alcoholism (IIPA) for enhancing self-regulation: Preliminary evidence | Included |
| Pinón-Blanco, et al., 2022^108^ | Efficacy of the Therapeutic Game “Trisquel” in the Treatment of Patients With Substance-Related Disorders Randomized Clinical Study | Context |
| Gamito et al., 2014a^87^ | Executive functioning in alcoholics followiing in mHealth cognitive stimulation program: randomized controlled trial | Included |
| Roehrich & Goldman, 1993^63^ | Experience-dependent neuropsychological recovery and the treatment of alcoholism. | Context |
| Berry et al., 2022^109^ | Feasibility, reliability and validity of a modified approach to goal attainment scaling to measure goal outcomes following cognitive remediation in a residential substance use disorder rehabilitation setting | Main outcome |
| Houben et al., 2011^110^ | Getting a Grip on Drinking Behavior: Training Working Memory to Reduce Alcohol Abuse | Context |
| Valls-Serrano et al., 2016^111^ | Goal Management Training and Mindfulness Meditation improve executive functions and transfer to ecological tasks of daily life in polysubstance users enrolled in therapeutic community treatment | Intervention |
| Tschuemperlin et a., 2018^112^ | Learning to resist the urge: a double-blind, randomized controlled trial investigating alcohol-specific inhibition training in abstinent patients with alcohol use disorder | Context |
| Guarriello et al., 2025^113^ | Mediation of beneficial effects of an alcohol-specific inhibition training on drinking of patients with alcohol use disorder: The role of cognitive demands and inhibitory performance | Sample (comorbidity) |
| Mathai, 1998^81^ | NEUROPSYCHOLOGICAL REHABILITATION OF ALCHOHOLICS : A PRELIMINARY REPORT | Include |
| Eberl et al., 2014 | Implementation of Approach Bias Re-Training in Alcoholism-How Many Sessions are Needed? | Context |
| Finn et al., 2023^115^ | The Effects of Working Memory Versus Adaptive Visual Search Control Training on Executive Cognitive Function | Context/sample |
| Wanmaker et al., 2018^83^ | The efficacy of a working memory training in substance use patients: A randomized double-blind placebo-controlled clinical trial | Include |
| Snider et al., 2018^85^ | Working Memory Training Improves Alcohol Users' Episodic Future Thinking: A Rate-Dependent Analysis | Include |
| Gamito et al., 2013^86^ | Executive functioning in alcoholics following in addicts following health mobile cogntive stimulation. Evidence from alcohol and heroin patients | Include |
| Kaag et al., 2018^116^ | A high working memory load prior to memory retrieval reduces craving in non-treatment seeking problem drinkers | Context/ sample |
| Oliveira et al., 2018^117^ | Cognitive stimulation of alcoholics throug VR-based Instrumental Activities of Daling Living | Intervention |
| Stein, et al., 2022^118^ | Alcohol-Specific Inhibition Training in Patients with Alcohol Use Disorder: A Multicenter, Double-Blind, Randomized Clinical Trial Examining Drinking Outcome and Working Mechanisms. | Missing main outcome/ sample (comorbility) |
