# Supplementary material for: Neuropsychological rehabilitation in executive deficits resulting from alcohol use disorder: systematic review of literature
Source: Front Psychol. 2026 Apr 29;17:1805577. doi: 10.3389/fpsyg.2026.1805577 (PMC13168111; doi:10.3389/fpsyg.2026.1805577)
Supplement: Supplementary file 2 [file Table_2.DOCX]

**Supplementary Material 2 - Table 1**

| **Reference** | **Methodology** | | | | | | | |
| --- | --- | --- | --- | --- | --- | --- | --- | --- |
|  | **Population/participants** | | | **Inclusion criteria** | **Exclusion criteria** | **Co-morbidity** | **Other consumptions - drugs** | **Participation rate** |
|  | **N analyzed** | **Age** | **Sex** |  |  |  |  |  |
| Rupp et al, 2012 | 37 | up to age 65 years; experimental group: mean age 45,2(SD=10.5) years; control group: mean age 45.5 (SD=8.8) years | Both | Diagnosis of alcohol dependence (DSM-IV ); Nonamnesic and nondemented ; standardized long-term (6- to 8-week) abstinencebased inpatient treatment at the Alcohol Dependence Treatment Unit of the Innsbruck Medical University; > 65 years old; one or more “mild” cognitive deficits in one cognitive tasks(attention/executive function) - performance score of at least 1 SD below the mean or the 16th percentile of provided norms of the neuropsychological tests used and criteria for mild neurocognitive disorder; | Mental disorder ( not depressive disorders); psychotropic medication (not antidepressant, anticonvulsant, or anticraving medication); substance dependence / substance abuse /recurrent use of illicit drugs within the last year (not cannabis; last intake > 1 month); neurological diseases and medical diseases (traumatic brain injury - loss of consciousness, head tumor, non-alcohol-related seizures);physical illnesses that interfere with neuropsychological assessment or rehabilitation | Not reported | Not reported | 4 not found mild cognitive deficit;4 lost follow-up ( control group): 1 tested positive for alcohol ; 3 dropped out of the study |
| Kumar et al, 2019 | 50 | 18- 45 years old;experimental group - mean age 34.28 (SD= 5.33)years; control group- mean age 34.08 (SD=5.73) | Male | Diagnosis of alcohol dependence (ICD-10); 18 to 45 years old early onset alcohol dependence (before 25 years of age), one or more first degree family members with alcohol dependence, | Other substance dependence (except tobacco) such as cannabis; major psychiatric disorders (schizophrenia, mood disorders; self-reported major co-morbid medical; neurological disorders); severe memory impairments (Mental State Examination- HMSE: less than 24]; Family history of psychiatric disorders (other than alcohol dependence) in first degree; clinical impression of mental retardation;  cognitive remediation/ meditation practices/ structured psychological therapy in past one year | No | Not reported | No |
| Gamito et al, 2013 | 61 | Mean age 45.64 (SD=9.67) years | Both | Male; a primary diagnosis of alcohol dependence | Not reported | Not reported | Heroin abuse | Not reported |
| Gamito et al, 2014b | 41 | mean age 45.45 years (SD=10,31); control group: mean age 48,61(SD=8.02) years; experimental group: mean age 41.62 (SD=11.4) years | Both | Diagnosis alcohol dependence (DSM-IV ); hospitalization private clinic ; scored higher than the cut-off - Mini Mental Examination Test; minimal computer literacy | Dependency from substances;history of previous neurological disorders | History of previous neurological - exclued | No | 4 lost to follow-up: 2 unable to atend; 1 unknown (experimental group); 1 unknown (conytrol group) |
| Gamito et al, 2014 a | 54 | Mean age 45.37years (45,37 SD= 10,12) | Both | Alcohol dependents fromalcohol-rehab clinic; scores above higher than the cut-off values - Mini Mental State Examination; no clinical scores on the Symptoms Checklist Revised; minimal computer literacy | Dependency on substances other than alcohol or with a history of previous neurological disorders; minimal computer literacy | History of previous neurological - exclued | No | 14 lost to follow-up: 1 refused; 6 unable to atend (experimental group); 1 moved; 5 unknown (control group) |
| Mathai et al, 1998 | 8 | 30- 45 years old | Male | Diagnosis of alcohol dependence (ICD-9); 30- 45 years old; abstinent for three weeks;  psychotropic drugs | Not report | No | No | Not reported |
| Wanmaker et al, 2018 | 74 | 16-70 years old; experimental group: mean age 38.53 years, control group: mean age 36.6 years | Both | Diagnosis of cannabis, cocaine, and/or alcohol dependence (DSM–IV–TR) | 16–70 years old; psychotic episode in the last month; severe brain damage; neurodegenerative disease; | Not reported | Cocaine, cannabis use disorder | Not reported |
| Snider  et al, 2018 | 31 | 18-65 years old; experimental group: mean age 42,5 (SD=2.0) years, control group: mean age 42,4 (SD=2.3) years | Both | Consumption in the last 6 months;More than 3 criteria for álcool dependence DSM-IV-TR | Not pregnant or lactating, not have any current significant medical or psychological disorders; history of stroke, seizures, loss of consciousness | History of stroke, seizures, loss of consciousness -exclued | Not reported | 18 initially excluded (3 inconsistent; 8 not inclusion criteria; 5 lost contact, 2 unknown); lost foloww-up: 14 experimental group (5 lost contact; 6 too mutch time beteween sessions; 3 voluntary withdraw)+ 18 control group (9 lost contact; 3 too mutch time beteween sessions; 1 voluntary withdraw; 3 unknow) |
