# Supplementary material for: Neuropsychological rehabilitation in executive deficits resulting from alcohol use disorder: systematic review of literature
Source: Front Psychol. 2026 Apr 29;17:1805577. doi: 10.3389/fpsyg.2026.1805577 (PMC13168111; doi:10.3389/fpsyg.2026.1805577)
Supplement: Supplementary file 3 [file Table_3.DOCX]

**Supplementary Material 3 - Table 2**

| **Characterization of Neuropsychological Rehabilitation** | | | | | | | | | | |
| --- | --- | --- | --- | --- | --- | --- | --- | --- | --- | --- |
| **Reference** | **Type/**  **Typology** | **Porpose of intervention** | **Cognitive tasks^1^** | **Other tasks** | **Strategies of intervention** | **Executive domains avaliables** | **Other cognitive domains** | **Number of sessions / duration (days,hours>)** | **Concomitant interventions** | **Control group** |
| Rupp et al, 2012 | Multidomains | Benefits of neuropsychological rehabilitation on cognitive functioning (Main objective: evaluate whether neuropsychological rehabilitation improves cognitive functioning (trained and untrained cognitive domains) Secondary objective: evaluate whether the benefits of NR generalize to other non-cognitive domains: craving and and  well-being) | Cognitive stimulation exercises ( executive functions, attention, memory; computer game-  “Towers of Hanoi)^1^ | Simple exercises for relaxing | Techonology | Working memory; Inhibition | Attention, memory, verbal fluency | 12 sessions/4 weeks/ 3 sessions per week (45–60 minutes per session) | Usual treatment | Usual treatment (standard treatment followed detoxifi cation treatment conducted in a unit different from that of the study) |
| Kumar et al, 2019 | Multidomains | Benefits of neuropsychological rehabilitation on clinical variables; (Main objetive: test the effectiveness of an executive function rehabilitation program secondary objectives: evaluate the benefits of RN in self-regulation) | Cognitive stimulation exercises | Qigong, Tai Chi Chuan exercises | Not report | Mental flexibility; working memory; inhibition; | Decision making | Sessions number- not report/18 days; | Usual treatment | Usual as treatment |
| Gamito et al, 2013 | Multidomains | Benefits of neuropsychological rehabilitation on cognitive functioning (to assess the efficacy of a rehabilitation program, based on a mobile device, improves executive functioning in recovery from PUA | Cognitive stimulation exercises/ serious games: attention, memory, decision making, language, processing speed, strategic planning, perception; working memory; logical reasoning exercises | No | Technology | Working memory, planning, cognitive flexibility | Attention,decision making, language, logical reasoning, speed of processing | 8-12 sessions/ 1 moth/ 2-3 sessions per weeek (starting from the sixth day of treatment) | Usual treatment | Usual treatment |
| Gamito et al, 2014b | Multidomains | Benefits of neuropsychological rehabilitation on cognitive functioning (Main objective: to assess the efficacy of a rehabilitation program, based on a mobile device, in recovery executive functioning in recovery) | Cognitive stimulation exercises (attention; working memory; logical reasoning exercises) | No | Technology/paper-and-pencil | Working memory; | Attention;logical reasoning | 10 sessions/ 4 weeks/ 3 sessions per week/ (50-60 minutes per session) | Not reported | Not reported |
| Gamito et al, 2014a | Multidomains | Benefits of neuropsychological rehabilitation on cognitive functioning (Main objective: assess the effects NR program, using mobile technology and serious games) | cognitive stimulation exercises: perception; processing speed; reasoning; attention; memory; decision making; planning; spatial vision tasks | No | Technology | working memory, planning | Attention,decision making | 10 sessions/4 weeks/ 2-3 sessions per week | Medication and vitaminis | Treatment as usual |
| Mathai et al, 1998 | Multidomains | Benefits of neuropsychological rehabilitation on cognitive functioning | Cognitive stimulation exercises: symbol-symbol substitution task under auditory distraction; digit cancellation task; verbal and visual memory tasks;  drawing test; Bhatia's performance test of intelligence; Koh's block design test of WAIS;porteus, matrix | No | Technology/paper-and-pencil | Planning; | Attention, information processing; memory; reasoning | 43 sessions/ 6 weeks / 1 session per day | Counselling sessions | Counseling once a week |
| Wanmaker et al, 2018 | One domain | Benefits of neuropsychological rehabilitation on clinical variables (analyze the efficacy of working memory training in craving) | Cognitive stimulation exercises: digit span; reading span task; stroop task; training paradigm; symmetry span; dual n-back task | No | Technology | Woking memory | No | 24 sessions/1 week/ (25 minutes per session) | Usual treatment - individual and group cognitive behavioral therapy | 0-back task (compare the stimuli with the first-seen stimuli) |
| Snider et al, 2018 | One domain | Benefits of neuropsychological rehabilitation on cognitive functioning (analyze the effect of working memory in performance on near-transfer and far-transfer delay discounting tasks) | Cognitive stimulation exercises - Cogmed® software (working memory training) | No | Techology | Working memory (verbal and visuospatial) | No | 20 sessions (not report) | Not reported | Memory training tasks without changing the degree of difficulty |

^1^ It was decided not to discriminate between cognitive and executive tasks
