# Supplementary material for: Neuropsychological rehabilitation in executive deficits resulting from alcohol use disorder: systematic review of literature
Source: Front Psychol. 2026 Apr 29;17:1805577. doi: 10.3389/fpsyg.2026.1805577 (PMC13168111; doi:10.3389/fpsyg.2026.1805577)
Supplement: Supplementary file 4 [file Table_4.DOCX]

**Supplementary Material 4 - Table 3**

| **Results of neuropsychological rehabilitation** | | | | | |
| --- | --- | --- | --- | --- | --- |
| **Reference** | **Transfer effect** | **Evaluation moments** | **Outcome measure** | **Cognitives outcomes** | **Other outcomes** |
| Rupp et al, 2012 | transfer effects:  noncognitive outcomes and untrained cognitive domains | Baseline assessment (start of inpatient treatment); folow-up: after NR (at the end of treatment - 4 weeks) | tests to assess executive functions: digit span - Wechsler Adult Intelligence Scale–Revised (WAIS-R); spatial Stroop task; Verbal fluency (phonemic - three letters;semantic -two categories); Trail Making Test Tests to assess another cognitive functions: Test Battery on Attentional Performance (TAP); ; California Verbal Learning Test; Munich Verbal Memory Test; Complex Figure Test; MehrfachwahlWortschatz-Test; Mini-Mental State Examination (MMSE); Block Design (WAIS-R) and CFT (CFT-copy); tests to evaluate clinical symptoms: Beck Depression Inventory (BDI); SCL90-R; Obsessive Compulsive Drinking Scale | Improvements with NR in working memory; not improvements in cognitive inhibition | Improvements with NR: psychological well-being and craving |
| Kumar et al, 2019 | Not reported | Baseline assessment (after 3–4 days of alcohol detox); follow-up: after 18 days of treatment; for six months to compare the abstinence | tests to assess executive functions: Test, Color Trails Test 1 and 2, Five- Point Test, Digit span, Spatial Span, Stroop color-word interference test, Game of Dice; Matrix Reasoning; Controlled Oral Word Association (COWA) tests to evaluate clinical symptoms: Task Affect Regulation Checklist (ARC), , | Improvements with NR: domains of executive functions; affect regulation | Improvements with NR: lower relapses and long term abstinence abstinence |
| Gamito et al, 2013 | Not reported | Baseline assessment (before NR); follow-up: 4 weeks (least 30 days between evaluations) | tests to assess executive functions:   Frontal Assessment Battery – FAB  Card Sorting Test - WCST; Color Trail Test; Tests to assess another cognitive functions: Mini Mental State Examination Test - MMSE | Improvements with NR: general cognitive abilities; executive functioning; ( group with mobile technologies) | Not reported |
| Gamito et al, 2014b | Not reported | Baseline assessment (before NR; first and fourth day after the screening); follow-up: 30 days after | tests to assess executive functions: Frontal Assessment Battery; Tests to assess another cognitive functions: Mini Mental State Examination Test - MMSE | Improvements with NR: Executive functions (only experimental group | Not reported |
| Gamito et al, 2014a | Not reported | Baseline assessment - after screening; follow-up: at least 30 days later | tests to assess executive functions: Frontal Assessment Battery – FAB; Wisconsin Card Sorting Test - WCST; Color Trail Test; Tests to assess another cognitive functions: Mini Mental State Examination Test - MMSE | improvements with NR: general cognitive abilities; mental flexibility; psychomotor processing speed; and attentional ability in both groups; greater enhancements in executive functions in the experimental group | Not reported |
| Mathai et al, 1998 | Effects transfer: speed of serial information processing; memory  Not efefcts transfer: family functioning; long term abstinence | Baseline assessment, follow-up: 6 weeks | tests to assess executive functions: NIMHANS neuropsychological battery Tests to assess another cognitive functions: digit symbol task of Wechler Adult; Intelligence Scale given with distraction (trafficnoise) for two minutes; The figure identification task of Weintraub & Mesulam (1986); task with the rhythm detection task (Misra & Rao, 1994); concept formation test (Rao, 1976) | Improvement with NR: information processing, memory | Not improvements with NR: family functioning and long term abstinence |
| Wanmaker et al, 2018 | Not reported | Baseline assessment; follow-up: 1 week | tests to assess executive functions: Digit Span; Reading Span Task; Stroop task; Training paradigm; Symmetry Span; Dual n-back task; tests to evaluate clinical symptoms: Mini International Neuropsychiatric Interview Plus; Addiction Severity Index; Desires for Alcohol Questionnaire; . Obsessive Compulsive Drug Use Scale; Obsessive Compulsive Drinking Scale; Barratt Impulsivity Scale–11; Beck Depression Inventory–Second Edition; State–Trait Anxiety Inventory; | Improvements on the trained tasks in both groups (more advancements control group); more improvements for three components in the intensive group. | Not improvements with NR: craving, substance use, impulsivity, attention bias, psychopathology |
| Snider et al, 2018 | Not efefcts transfer: family functioning; long term abstinence | Baseline assessment; follow-up: after NR (pre- and post- training) | tests to assess executive functions: Near-transfer Task– Following Instructions;Far-transfer Task – Delay Discounting Alone; tests to evaluate clinical symptoms: Audit | Improvements with NR: performance on near-transfer task (experimental group) | Not reported |

Subtitle: NR - Neuropsychological Rehabilitation
